# Supplementary material for: Supraphysiological protection from replication stress does not extend mammalian lifespan
Source: Aging (Albany NY). 2020 Apr 6;12(7):5612–24. doi: 10.18632/aging.103039 (PMC7185120; doi:10.18632/aging.103039)
Supplement: Supplementary Figures [file aging-12-103039-s001..pdf]

SUPPLEMENTARY FIGURES

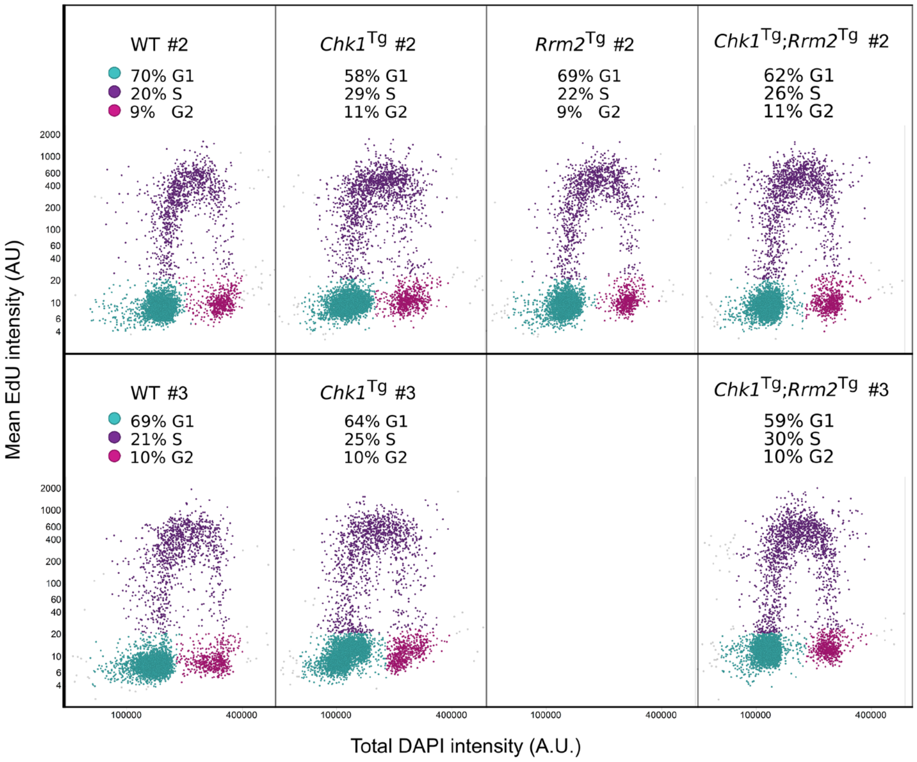

Supplementary Figure 1. *Chk1*^{Tg}, *Rrm2*^{Tg} and *Chk1*^{Tg};*Rrm2*^{Tg} MEFs replicate similarly to WT MEFs. Cell cycle distribution of MEFs determined by EdU incorporation and DAPI profiles, as in figure 1D but with two additional MEF lines for WT, *Chk1*^{Tg} and *Chk1*^{Tg};*Rrm2*^{Tg} MEFs, and one for *Rrm2*^{Tg} MEFs, derived from another mating. The MEF lines in this figure are all derived from the same mating. At least 7000 cells from two technical replicates were quantified per condition using high-content microscopy.

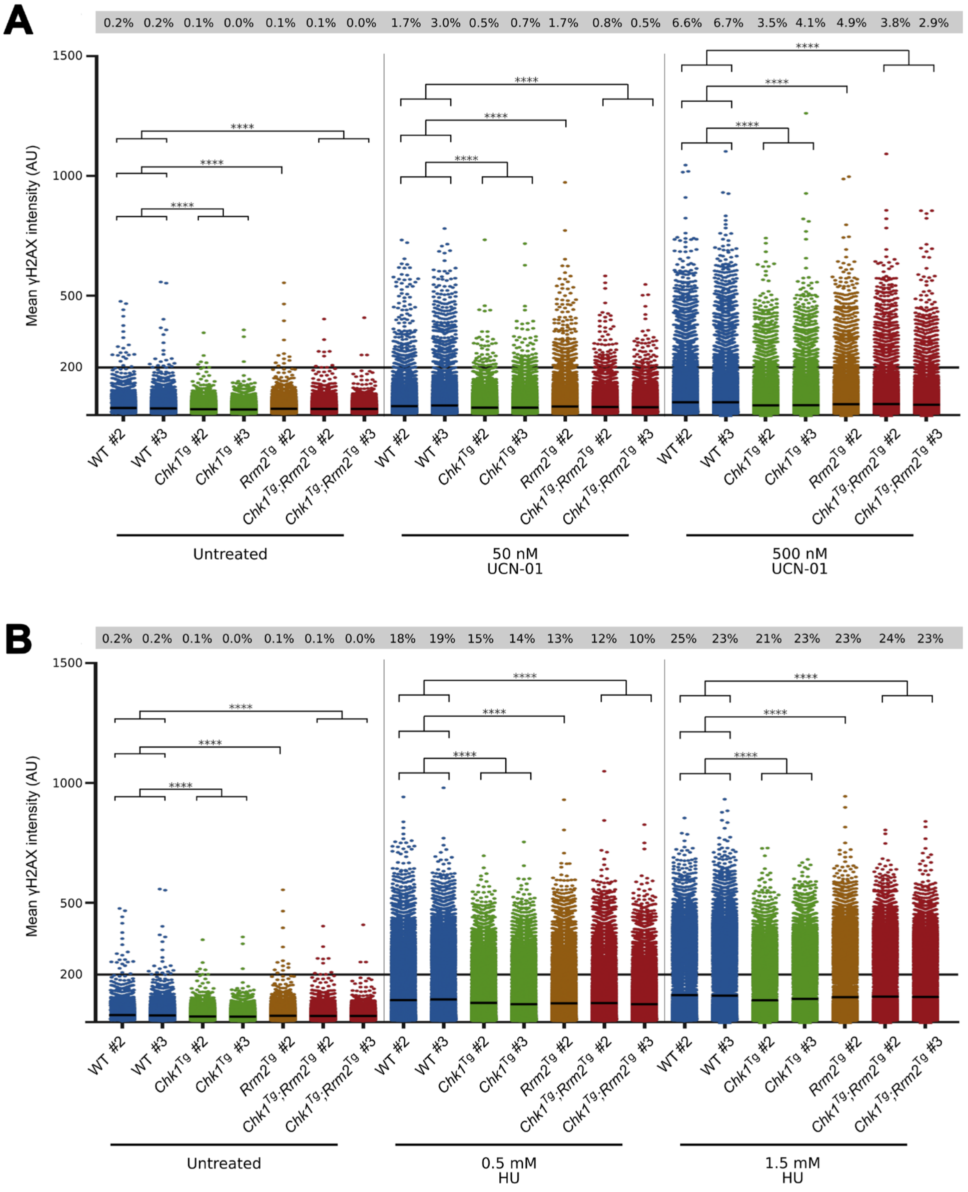

Supplementary Figure 2. *Chk1*^{Tg}, *Rrm2*^{Tg} and *Chk1*^{Tg};*Rrm2*^{Tg} MEFs are more resistant to induction of replication stress compared to WT MEFs. Quantification of γH2AX intensity in MEFs treated with UCN-01 (**A**) or HU (**B**) at indicated concentrations for four hours, as in Figure 1E–1F but with two additional MEF lines for WT, *Chk1*^{Tg} and *Chk1*^{Tg};*Rrm2*^{Tg} MEFs, and one for *Rrm2*^{Tg} MEFs. At least 10000 cells obtained from two technical replicates were quantified per condition using high-content microscopy. Percentages above graphs indicate cells with γH2AX intensity above a threshold of 200 AU, and means are indicated by horizontal black lines for each condition. The control cells are the same for (**A**) and (**B**), as the results were obtained from the same experiment. **** = P ≤0.0001. Statistical significance was determined using an unpaired t-test, by pooling the cells with the same genotypes as indicated.
